# Spread of the novel vancomycin-resistant *Enterococcus faecium* strain ST1299/vanA from local level in Germany to cross-border level in Austria, 2018 to 2022

**DOI:** 10.2807/1560-7917.ES.2025.30.20.2400389

**Published:** 2025-05-22

**Authors:** Anca Rath, Bärbel Kieninger, Nilufarbayim Mirzaliyeva, Guido Werner, Jennifer K Bender, Martin A Fischer, Adriana Cabal-Rosel, Werner Ruppitsch, Helena MB Seth-Smith, Adrian Egli, Milo Halabi, Anna Hörtenhuber, Yarub Salaheddin, Wolfgang Prammer, Heidrun Kerschner, Rainer Hartl, Martin Ehrenschwender, Andreas Ambrosch, Jörn Kalinowski, Levin Joe Klages, Christian Rückert-Reed, Tobias Busche, Alexander Kratzer, Aila Caplunik-Pratsch, Anja Eichner, Jürgen Fritsch, Wulf Schneider-Brachert

**Affiliations:** 1 Department of Infection Prevention and Infectious Diseases, University Hospital Regensburg, Regensburg, Germany; 2 Department of Infectious Diseases, Division of Nosocomial Pathogens and Antibiotic Resistances, Robert Koch Institute, Wernigerode, Germany; 3 Division for Public Health, AGES – Austrian Agency for Health and Food Safety, Vienna, Austria; 4 Division for Public Health, AGES – Austrian Agency for Health and Food Safety Institute for Medical Microbiology & Hygiene, Graz, Austria; 5 Institute for Medical Microbiology, University of Zurich, Zurich, Switzerland; 6 Institute for Clinical Pathology, Microbiology and Molecular Diagnostics, Barmherzigen Schwestern Ried Hospital, Ried, Austria; 7 Institute of Pathology, Upper Austrian Health Holding GmbH, Pyhrn-Eisenwurzen Clinical Centre Kirchdorf Steyr, Steyr, Austria; 8 Institute for Hygiene and Microbiology, Wels-Grieskirchen Hospital, Wels-Grieskirchen, Austria; 9 National Reference Centre for Antimicrobial Resistance, Institute for Hygiene, Microbiology and Tropical Medicine, Ordensklinikum Linz Elisabethinen, Linz, Austria; 10 Institute of Laboratory Medicine, Microbiology and Infection Prevention, Hospital of the Merciful Brothers, Regensburg, Germany; 11 Centre for Biotechnology (CeBiTec), Bielefeld University, Bielefeld, Germany; 12 Medical School East Westphalia-Lippe, Bielefeld University, Bielefeld, Germany; 13 Hospital Pharmacy, University Hospital Regensburg, Regensburg, Germany

**Keywords:** VRE, ST1299, WGS, emergence of pathogens, genome-oriented surveillance

## Abstract

**Introduction:**

Vancomycin-resistant *Enterococcus faecium* (VREfm) isolates of sequence type (ST)1299 were described recently in south-eastern German hospitals and rapidly expanded from local to cross-border level.

**Aim:**

We describe the spread of the novel VREfm strain ST1299/vanA on a genetic, geographical and temporal level during the first 5 years after its detection.

**Methods:**

At University Hospital Regensburg (UHoR), routine VREfm surveillance is whole genome sequencing-based (≥ 1 VREfm per *van*-genotype, patient and year). In this observational cohort study, we analysed one VREfm ST1299 isolate from our database (2016–2022) per patient and year. Isolates were added from the Hospital of the Merciful Brothers Regensburg (MBR), the National Reference Centre for Staphylococci and Enterococci (NRC), and clinical isolates from Austria.

**Results:**

We identified 635 VREfm ST1299 isolates (100% *vanA*), including 504 from Regensburg, and 113 blood cultures. ST1299 isolates were first detected in 2018 simultaneously in Regensburg (n = 2) and southern Bavaria (n = 2), with local (UHoR) and regional numbers increasing rapidly from 2020, shifting to national scale in the same year. Genome data, analysed by cgMLST, showed a predominance of ST1299/CT1903 (315/504 isolates, 62.5%) and ST1299/CT3109 (127/504 isolates, 25.2%) isolates from Regensburg. By 2021, ST1299/CT1903 reached Upper Austria causing hospital outbreaks (n = 5). Phylogeny analysis suggests common ancestors with VREfm ST80, ST18 and ST17.

**Conclusion:**

Since their emergence in 2018, two highly transmissible subtypes of ST1299/*vanA* reached national, then cross-border scale. The observed outbreak tendency may explain the rapid and successful spread and the high clonality in our collection.

**Key public health message**

**What did you want to address in this study and why?**

Enterococci are intestinal bacteria that can cause infections in certain patient populations (i.e. immunocompromised patients). Due to limited therapy options, it is essential to prevent the spread of multidrug-resistant variants like vancomycin-resistant *Enterococcus faecium* (VREfm). We investigated the spread of a novel subtype, ST1299/*vanA,* seen in south-eastern German hospitals in recent years, and its rapid expansion to cross-border level.

**What have we learnt from this study?**

Molecular analysis of all VREfm ST1299/*vanA* isolates detected in Regensburg, Germany, since its emergence in 2018 showed that the genetic diversity among isolates remained low during the 5-year period, whereas transmissions and clinical outbreaks were frequent. In particular, two subtypes, CT1903 and CT3109, caused the rapid spread in Regensburg and also at a national level in Germany and Austria between 2018 and 2022.

**What are the implications of your findings for public health?**

Making decisions on infection prevention policies requires continuous analysis of local epidemiology at a molecular level. Our data clearly shows that during early stages of VREfm spread, the high genetic similarity of isolates complicates differentiating between outbreaks and background epidemiology. Thus, more molecular surveillance data are needed for understanding how VREfm spreads in healthcare facilities.

## Introduction

Enterococci are Gram-positive cocci that occur naturally in the intestines of humans and animals [1]. *Enterococcus faecium* causes nosocomial bloodstream infections (BSI) or urinary tract infections in critically ill patients [2]. Due to intrinsic resistance to several antibiotics including beta-lactam antibiotics, few therapeutic options are available and vancomycin is often used as first choice treatment. Moreover, vancomycin-resistant *E. faecium* (VREfm) strains are associated with increased mortality in patients in intensive care units (ICUs), haematology departments or post transplant [3]. The burden of disease is highest in males, those younger than five years or older than 55 years [4,5]. Thus, the emergence and rapid spread of VREfm has led to major concerns regarding future availability of therapeutic options [6,7]. European Antimicrobial Resistance Surveillance Network (EARS-Net) reported a particularly high proportion of VREfm among *E. faecium* isolates from clinical samples in Germany (2019: 26.3% (n = 2,797), 2021: 21.6% (n = 4,721)) compared with in Europe (2019: 18.3% (n = 16,523), 2021: 17.2% (n = 22,315)) [8,9]. The Bavarian antibiotic resistance database (BARDa) presents similar data for VREfm among *E. faecium* (2019: 39.3% (n = 857), 2022: 26.6% (n = 1,970)) [10]. In neighbouring countries such as Austria and Switzerland, VREfm is less frequently seen (Austria 2019: 3.2% (n = 537), 2021: 2.0% (n = 697)*;* Switzerland 2019: 1.8% (n = 399), 2021: 1.9% (n = 573)) [8]. Although the prevalence of VREfm varies greatly across Europe, its burden of disease among Europeans was recently estimated as an increase in attributable deaths of 1.95 between 2007 and 2015 and median disability-adjusted life years (DALY) of 5.49 years [4]. Countries with the highest prevalence of VREfm are Ireland, Italy, Greece, Cyprus, Portugal and Poland [4]. This is particularly concerning due to contrasting literature on efficiency of infection prevention and control (IPC) measures and lack of consensus regarding proper clinical management [11,12]. Therefore, much effort is currently invested in understanding the dynamics and spreading patterns of VREfm.

At the University Hospital in Regensburg (UHoR), Bavaria, Germany, VREfm was first detected in 2004 and, until 2019, the local (Regensburg City) epidemiology was dominated by the glycopeptide-resistance genotype *vanB* (VRE/*vanB*) [13,14]. This corresponded to data from the German National Reference Centre for Staphylococci and Enterococci (NRC) at the Robert Koch Institute in Wernigerode, which has recorded the predominance of VRE/*vanB* in Germany since 2006 [15,16]. However, in 2020, VRE/*vanA,* which was first detected at UHoR in 2018, reached a proportion of 42.7% (138/319) of VREfm isolates analysed at UHoR. Using whole-genome sequencing (WGS) as part of genome-oriented IPC policy at UHoR, we detected that a novel strain – sequence type (ST)1299/*vanA* – contributed to the increase in VRE/*vanA,* reaching a proportion of 28.8% (93/319) [14]. Moreover, clonality seen during 2020 was high. Clusters defined by a difference of ≤ 3 alleles in pairwise comparison using cgMLST comprised up to 18 strains. To the authors’ knowledge, to date only two additional study groups from Bavaria, Germany, have reported cases of ST1299/*vanA*, including from samples of wastewater [17-19]. Internationally, clinical ST1299/*vanA* isolates were reported from Austria (2022–2023, 57 cases), Denmark (2017, one case), Iran (2021, one case) and Sweden (2022, 17 cases) [18,20-22]. The NRC also noted increasing proportions of VRE/*vanA* (2021: 31.3% of 686, 2022: 45.3% of 400). In contrast to the data obtained from Bavaria, VRE/*vanA* isolates sent to the NRC from other German microbiology laboratories are most often attributed to the lineages ST80/ complex type (CT)1470 and ST117/CT929, suggesting that VREfm ST1299/*vanA*, in particular CT1903 and CT3109, may have emerged in Bavaria [15].

Given the rapid spread of VREfm ST1299/*vanA* within UHoR, questions arose regarding how this strain had propagated since its first detection in 2018 up until December 2022 [17,18]. Thus, we aim to retrace its steps by analysing a unique VREfm ST1299/*vanA* isolate collection from Austria and Germany [20]. By summarising the available data, we intend to grasp the epidemic potential of this highly transmissible lineage and gain information that will enable us to prevent similar chains of propagation in the future.

## Methods

### Study design

We report the results from our retrospective observational study according to the Strengthening the Reporting of Observational Studies in Epidemiology (STROBE) guidelines [23]. Isolates of all patients diagnosed with VREfm classified as ST1299/*vanA* at UHoR, the Hospital of the Merciful Brothers Regensburg (MBR), the NRC and the Austrian Agency for Health and Food Safety (AGES) during WGS-based surveillance or outbreak management between 2018 and 2022 were included.

At the initiating facility, UHoR, the IPC policy for VREfm includes contact precautions, such as patient isolation. Screening for VREfm is performed systematically in high-risk departments (oncology, ICUs), and is dependent on the risk evaluation of the attending physician in other departments. During the COVID-19 pandemic, however, criteria were inconsistent and are not retraceable retrospectively. Additionally, an antibiotic stewardship team is available and performs weekly visits to ICUs and high-risk departments. The consumption of antibiotics as retrieved from the ADKA-If-DGI project (a project in cooperation of the Federal Association of German Hospital Pharmacists (ADKA), the Department for Infectious Diseases at the University Hospital Freiburg (If), and the German Society for Infectiology (DGI)) and VREfm incidence are depicted in Table 1.

**Table 1:** Characteristics of ST1299/*vanA* vancomycin-resistant-positive *Enterococcus* patients at University Hospital Regensburg, the Hospital of the Merciful Brothers Regensburg, the German National Reference Centre^a^, and the Austrian Agency for Health and Food Safety during whole-genome sequencing-based surveillance or outbreak management, 2018–2022

VREfm isolates obtained from		n					
UHoR		460					
MBR		43					
NRC		89					
AGES		43					
**Age (years; UHoR and MBR only)**							
Median (range; IQR)		68 (3–95; 19)					
**Sex (Regensburg only)**		**n**		**%**			
Male		293		58.3			
Female		210		41.7			
**Sample type (Regensburg only)**							
**Obtained from**	**Blood culture**	**Urine**	**Bile**	**Screening**	**Other clinical sample type^b^**		**Unknown**
UHoR	22	102	5	277	54		0
MBR	4	14	0	16	8		1
NRC	69	4	0	6	4		6
AGES	18	8	0	3	5		9
**Hospital department (isolates from UHoR and MBR only)**							
**Department**		**n**					
Gastroenterology		68					
Haematology/oncology		65					
Cardiology/pneumology		45					
Nephrology		34					
General surgery		74					
Surgical, other		72					
Interdisciplinary department or other medical field		122					
Unknown		23					
ICU/IMC		140					
**Year**		**2018**	**2019**		**2020**	**2021**	**2022**
**VREfm incidence density per 1,000 hospital days at UHoR**							
Total		2.7	2.7		3.1	4.3	3.9
Nosocomial		0.7	0.9		0.9	1.9	1.2
**VREfm screening per 100 admissions at UHoR**							
Screenings		5.6	5.1		5.8	9.5	5.7
**Antibiotic consumption in RDD per 100 hospital days at UHoR**							
Third generation cephalosporins		3.5	4.2		4.2	4.4	4.6
Carbapenems		6.6	6.4		7.0	8.9	8.4
Glycopeptides		5.3	5.6		6.1	7.1	7.0

AGES: Austrian Agency for Health and Food Safety; ICU: intensive care unit; IMC: intermediate care; IQR: interquartile range; MBR: Hospital of the Merciful Brothers Regensburg; NRC: German National Reference Centre for Staphylococci and Enterococci; RDD; recommended daily doses; UHoR: University Hospital in Regensburg; VREfm: vancomycin-resistant *Enterococcus faecium.*

^a ^German National Reference Centre for Staphylococci and Enterococci, Robert Koch Institute, Wernigerode.

^b^ Including wound swabs, intraoperative samples and respiratory materials.

#### Isolate selection

The isolate selection criteria for sequenced isolates differed at participating facilities for various reasons and are described below. Data provided for further investigation included date of collection, city of collection and specimen type, where available. For isolates from Regensburg, Germany, further data were included (Table 1).

At the tertiary care hospital UHoR, a trained medical laboratory assistant assessed all VREfm-positive samples sent to the clinical microbiology laboratory for different VREfm morphotypes regardless of sample type or isolation unit/hospital. *E. faecium* was identified through mass spectrometry (Bruker microflex, Mannheim, Germany) and VREfm via in house *vanA/B*-PCR and stored at -80 °C (for samples from 2016 and later) [24]. Prior to 2016, only one isolate per patient and year was collected.

For the periods 2004–2010 and 2016–2019, one VREfm isolate per patient and year was sequenced for molecular surveillance purposes. Between 2011 and 2015, isolates were only sequenced in exceptional cases. One ST1299/*vanA* isolate per year and patient (where available) from the UHoR database up until December 2022 was included in the study regardless of specimen type.

All VRE/*vanA* isolates from the strain collection of the MBR’s microbiology laboratory between 2018 and 2022 were identified retrospectively. These isolates were sent to the outbreak laboratory of the UHoR for WGS. One VREfm ST1299/*vanA* isolate per patient and year identified in this collection until December 2022 was included in the study regardless of specimen type.

In Germany, Enterococci samples from any microbiological laboratory are sent voluntarily to the NRC for further investigation, including WGS. Whole-genome sequencing is performed systematically for isolates from blood cultures (BC) for the purpose of genomic surveillance activities or for outbreak investigations. All isolates (n = 89) typed as ST1299/*vanA* during outbreak investigations between 2016 and 2022 are included in the study.

All ST1299/*vanA* isolates from Upper Austria identified at AGES between 2021 and 2022, as previously reported [20], were included in the study regardless of specimen type or sequencing purpose.

#### Whole-genome sequencing

Whole-genome sequencing was performed at UHoR for all isolates from UHoR and MBR, 55 isolates from the NRC and 34 isolates from AGES either on a NextSeq device (Illumina Inc., Berlin, Germany), or, for some UHoR isolates collected between 2018 and 2021, MiniSeq device (Illumina Inc.) according to the manufacturer’s recommendation, acquiring 2x150 base pair (bp) reads using a mid-output cassette as previously described [14]. Of the isolates from the NRC, 35 were sequenced in domo on a NextSeq device according to the manufacturer’s recommendations and generating 2x150 bp reads except for samples UW22368, UW21208, UW18832, UW18830 and UW20224. These samples were sequenced on a MiSeq device (Illumina Inc.) generating 2x300bp reads (for UW20224 2x250bp). Nine isolates from Upper Austria were sequenced (2x150bp) by AGES on a NextSeq 2000 device. All facilities used the Nextera XT DNA library preparation kit (Illumina Inc.). A minimum average coverage of 50 (median: 129, interquartile range (IQR): 63) was achieved for all isolates.

**Figure 1:**
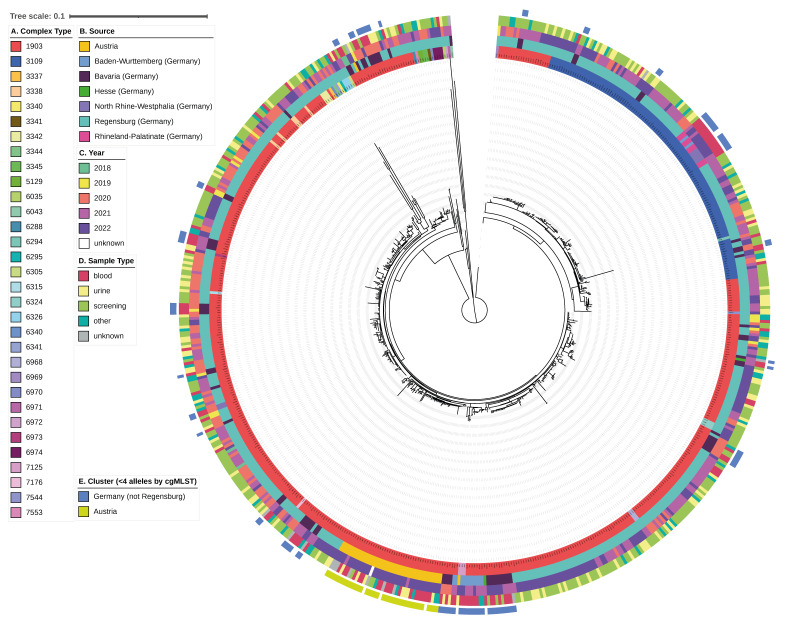
Core gene neighbour-joining tree of ST1299/*vanA* isolates, Germany and Austria, 2018–2022

Isolate D6593 (Bavaria, 2018; Figure 1) was also sequenced by Oxford Nanopore Technologies (ONT, Oxford, United Kingdom) as per manufacturer instructions using the ligation sequencing kit V14 and Prometheon R10.4.1 flowcells. A hybrid assembly of ONT and Illumina data was generated in unicycler version 0.5.0 (https://github.com/rrwick/Unicycler) resulting in a circular chromosome of 2.7Mb and 4 circular plasmid contigs.

The metadata are depicted as concentric rings from inside (circle A) to outside (circle E). The overall clonality was high with predominance of 2 subtypes – CT1903 (red) and CT3109 (blue) – as shown in circle A. Clusters with differences of ≤ 3 alleles in pairwise comparison by cgMLST (circle E) were frequently detected in isolates from the same German hospital (circle E: blue). Moreover, different clusters coexisted in Bavaria during the observed period. Among isolates from Austria (circle B: orange), 40 of 42 are assigned to the same cluster (circle E: green) and are genetically closely related (≥ 3 alleles by cgMLST) to isolates from Upper Bavaria. The colour-coding is described in the legend.

Comparative data analysis was performed at UHoR after assembling with SKESA version 2.4.0 [25] using multilocus sequence typing (MLST) and core-genome (cg) MLST with SeqSphere + software version 9.0.1 (Ridom, Muenster, Germany) using default parameters to determine ST and CT [26]. Clusters were defined as ≤ 3 alleles by cgMLST [26,27]. The core genome neighbour-joining tree was generated using SeqSphere + and metadata were superimposed using iTOL version 6.9 [28]. Determinants for resistance to linezolid were determined using LRE-Finder for all isolates from blood culture [29].

#### Origins of VREfm ST1299/*vanA*

We attempted to determine the phylogeny of VREfm ST1299/*vanA* using two different approaches.

Comparison of the D6593 hybrid assembly was performed against the 3,888 genomes available at https://pubmlst.org/organisms/enterococcus-faecium using all available loci and visualised in GrapeTree [30,31].

The hybrid chromosome assembly of D6593 was used as the reference against which to map all Illumina data using snippy version 4.6.0 (https://github.com/tseemann/snippy). The resulting alignment was analysed by Gubbins version 3.3.0 to identify and remove recombinations, and BactDating version 1.1.1 was used to reconstruct a dated phylogeny in triplicate with 1 million iterations using the arc model [32,33].

Twenty-four non-ST1299/*vanA* isolates belonging to STs and CTs frequently detected at UHoR (data not shown) before 2020 were mapped against a hybrid assembly of D6593.

Single nt polymorphisms (SNP) were determined using snippy and are provided as supplementary data [34]. The phylogram including the closest related isolates was generated using the Type Strain Genome Server (TYGS, https://tygs.dsmz.de) and the dDDH method [35], and edited with InkSkape version 1.3.2 (https://inkscape.org).

Calculations including proportions, medians, IQR, confidence intervals (CI), incidence and screening frequency were performed using Microsoft Excel.

## Results

### Isolate collection

In total, 635 VREfm ST1299/*vanA* isolates were identified from the participating institutions (clinical microbiology at UHoR: n = 460 including patients at UHoR (n = 343) and other local hospitals (n = 117), MBR (n = 43), NRC (n = 89) and AGES (n = 43)). Most isolates in this study were collected in southern Germany (577 of 635 isolates from Bavaria and Baden-Wuerttemberg including 74 of 89 isolates from the NRC).

All isolates were *vanA*-positive. No *vanB*-positive strains were detected. Further predicted resistance from genotypes is summarised in Table 2.

**Table 2:** Distribution of resistance determinants and mutations of vancomycin-resistant *Enterococcus faecium* isolates, Germany and Austria, 2018–2022

Resistant to	Genotype	%	n
Glycopeptides	*vanA*	100	635
Aminoglycoside	*aac(6')-I / aph(3')-IIIa*	20.6	131
	*aac(6')-I*	79.5	505
	*aac(6')-I / aac(6')-Ie/aph(2”)-Ia / aph(3')-IIIa*	0.3	2
Lincosamide	*cfr(B)*	1.4	9
	Subgroup analysis of isolates from blood cultures (n = 113): 23S rRNA mutations, *optrA, cfr, cfr(B), poxtA*	0	0
Erythromycin	*erm(B)*	70.2	446
Quinolone	*gyrA_S83Y/parC_S80I*	1.7	11
	*gyrA_S83I/parC_S80I*	98.7	627

### Timeline and spreading patterns

**Figure 2:**
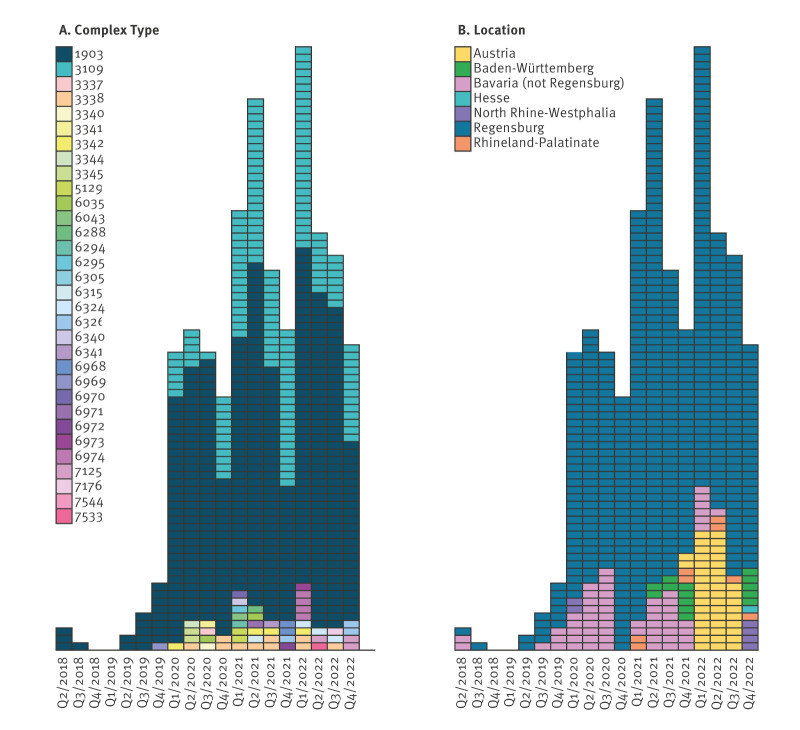
Timeline of A) complex type and B) geographical distribution of vancomycin-resistant *Enterococcus faecium* ST1299/*vanA* cases, Germany and Austria, 2018–2022

Q1: first quarter (1 January­–31 March); Q2: second quarter (1 April–30 June); Q3= third quarter (1 July–30 September); Q4: fourth quarter (1 October–31 December).

Molecular surveillance at UHoR identified the first two ST1299/*vanA* isolates in April 2018 (Figures 1 and 2).

A. ST1299/*vanA* was first detected in 2018 at University Hospital Regensburg and another Bavarian city. Starting in 2020, a rapid increase of ST1299/*vanA* isolates and the emergence of different complex types (CTs) was observed. Two subtypes, CT1903 and CT3109, predominated throughout the observed period.

B. The rise in ST1299/*vanA* cases was associated with a wider geographical expansion. Throughout the studied period, a growing number of ST1299/*vanA* were collected from blood cultures, as can be seen by the number of German (not Regensburg) isolates provided by the German National Reference Centre for Staphylococci and Enterococci. By the end of 2021, vancomycin-resistant *Enterococcus faecium* ST1299/*vanA* were seen in Upper Austria in association with clinical outbreaks.

Concomitantly, the NRC detected a further two isolates in southern Bavaria, Germany. During 2019, only 16 isolates were found, all identified regionally in Regensburg County in eastern Bavaria. By 2020, however, numbers had strongly increased and continued to increase during 2021 due to several outbreaks in regional hospitals including UHoR (see [36], data not shown). Isolates sent to the NRC also showed high clonality within the same centre and year (up to 22 alleles by cgMLST) (Figure 1).

Although WGS-reliant surveillance is not mandatory in Austria and is only performed voluntarily, VREfm ST1299/*vanA* was detected during investigations of clinical outbreaks (five outbreaks involving 3–16 patients) in Upper Austria, starting in December 2021. Due to its lagging detection compared with Germany, and close genetic relatedness (≤ 3 alleles by cgMLST) to isolates from Upper Bavaria and Regensburg (Upper Palatinate in Eastern Bavaria), we assume that one or more cross-border transmissions may have occurred.

#### Distribution of complex types

Although 32 different CTs were detected in our ST1299/*vanA* collection, only two were particularly common: CT1903 (430/635 isolates, 67.7%) and CT3109 (151/635 isolates, 23.8%). We assume that both strains spread regionally (CT1903) or locally (Regensburg City) (CT3109) between 2018 and 2019. The first and only type of ST1299/*vanA* detected in Germany until October 2019 was CT1903, dominating almost throughout the investigation period (with the exception of January–March 2022). Its cross-border spread to Upper Austria was then noticed due to outbreaks in 2021.

On the other hand, CT3109 was first detected in Regensburg in January 2020, and overall, only 18 isolates were detected more than 15 km away from the City of Regensburg. These were collected primarily in northern Bavaria, the Rhinehessen region and the Rhine-Ruhr Metropolitan region. Although CT3109 outbreaks were not seen between 2016 and 2022, CT1903 was observed. This subtype spread regionally and nationally in 2021. Up until December 2022, no CT3109 isolate had been reported beyond German borders.

Only 54 isolates, including one from Austria, were assigned to other CT (Figures 1 and 2). Among them, CT3338 was the most common (8 isolates).

**Figure 3:**
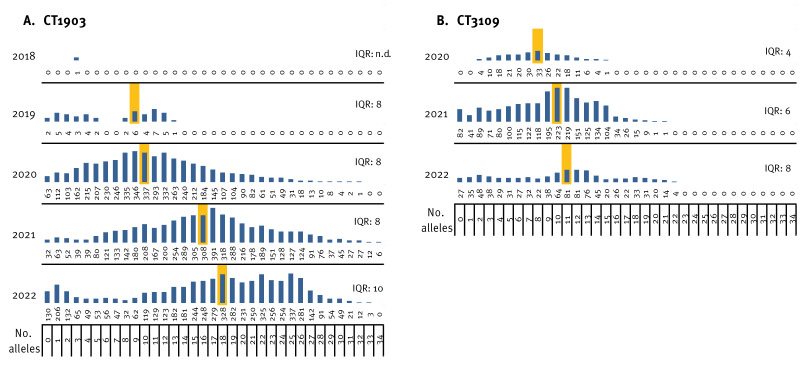
Allelic differences according to pairwise comparison of isolates for A) CT1903 and B) CT3109 collected at University Hospital Regensburg and the Hospital of the Merciful Brothers Regensburg, Germany, 2018–2022

Pairwise isolate comparison by cgMLST uncovered low local and regional diversity of the isolates throughout the study period. Among the 313 VREfm ST1299/CT1903/*vanA* isolates, a maximum difference of 37 alleles was found with a median of 16 (IQR: 9). The median difference increased by 1–6 alleles per year (Figure 3).

IQR: interquartile range; n.d.: not determined.

The X-axis of each graph is the number of isolates, the number below each bar delineates the number of allelic differences of pairwise compared isolates. Isolates collected at University Hospital Regensburg and the Hospital of the Merciful Brothers Regensburg during the same year differ by a maximum of 34 (CT1903) and 22 (CT3109) alleles per year. Allelic diversity can be seen to increase every year: the progression of the median allelic difference by cgMLST was 1–6 per year (orange box).

The largest shift of the median allelic difference was seen during 2021. Notably, outbreaks caused by different subtypes were observed in Regensburg City (data not shown). Concordantly, while different strains circulated locally in other German cities, local or regional clusters (≤ 3 alleles by cgMLST) were detected. Further, isolates from the same centre and year varied by up to 22 alleles (Figure 1).

In Austria, ST1299/*vanA* was initially identified during outbreak-related sequencing, where a median difference of 2 alleles was seen between ST1299/CT1903/*vanA* isolates from the same hospital (Figure 1) [20]. However, the overall median difference between Austrian isolates was 20 alleles. Nonetheless, all but two Austrian isolates were assigned to the same cluster (Figure 1).

For CT3109, which likely emerged after CT1903, there was a maximum difference of 23 alleles (median: 10 alleles) and a more stable median progression of two (2020–2021) and one (2021–2022) allele per year, respectively. To date, the authors have not detected outbreaks of CT3109 using the cluster definition described above.

#### Origins of ST1299/*vanA*

**Figure 4:**
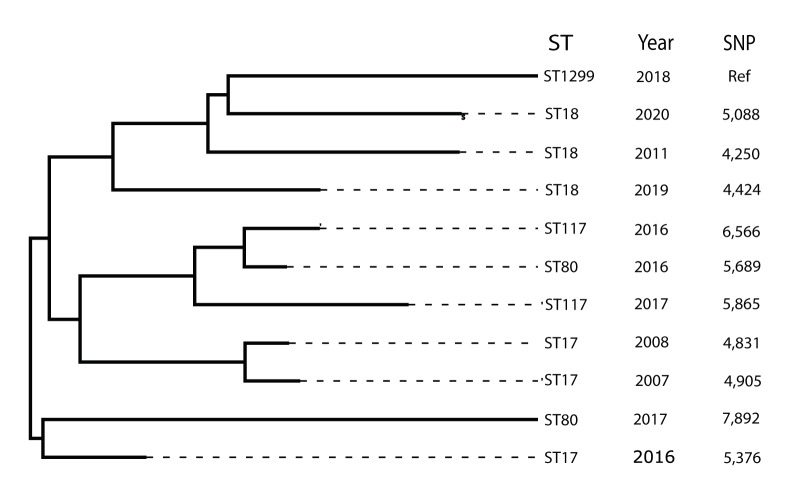
Phylogram of ST1299/*vanA* putative ancestral strains from the University Hospital Regensburg database, Germany, 2004–2020

SNP: single-nucleotide polymorphism; ST: sequence type.

Comparison of D6593 as reference genome to endemic local isolates detected an ST992/CT7179 isolate from 2016 as the closest relation (difference of 273 alleles and 6,495 SNP, respectively) (Figure 4 and Supplementary Table S1). In contrast, two ST17 and two ST18 isolates, that differed by over 300 alleles, showed differences of fewer than 5,000 SNP in a pairwise comparison, the nearest being 4,250 SNP. The remaining 21 isolates used for comparison belonged to ST78, ST80, ST117, ST186, ST192, ST202, ST208, ST721, ST780, ST1478, and differed by 5,088 to 7,892 SNP and between 303 and 402 alleles, respectively.

Of a total of 24 strains frequently detected at University Hospital Regensburg between 2004 and 2020, only four differed by < 5,000 SNPs from the ST1299/*vanA* reference strain from 2018. ST18 isolates were most closely related to ST1299/*vanA*, differing by only 4,250 and 4,424 SNPs, respectively.

**Figure 5:**
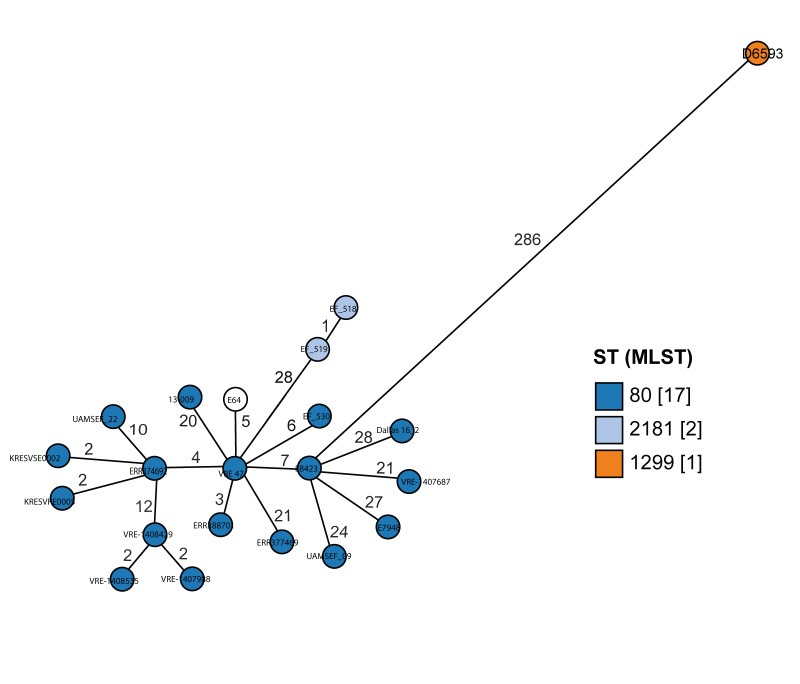
GrapeTree rendering of the PubMLST comparison of genome D6593 against the genome collection using all available loci

Comparison of D6593 with a broader range of isolates, namely the PubMLST Genome database, found the closest isolates to be from within ST80, albeit at a distance of 283 alleles (Figure 5). Concordantly, on an MLST level, ST1299/*vanA* and ST80 only differed by 1 SNP in the *purK* gene – namely 313G > A. However, five other less common STs also differed by 1–2 SNP in *purK* only (see Supplementary Table S2).

Only the closest cluster is depicted for clarity. The text in the circles indicate the isolate identity as provided by the PubMLST database. The numbers on the lines indicate the numbers of differing alleles according to pairwise comparison by cgMLST.

**Figure 6:**
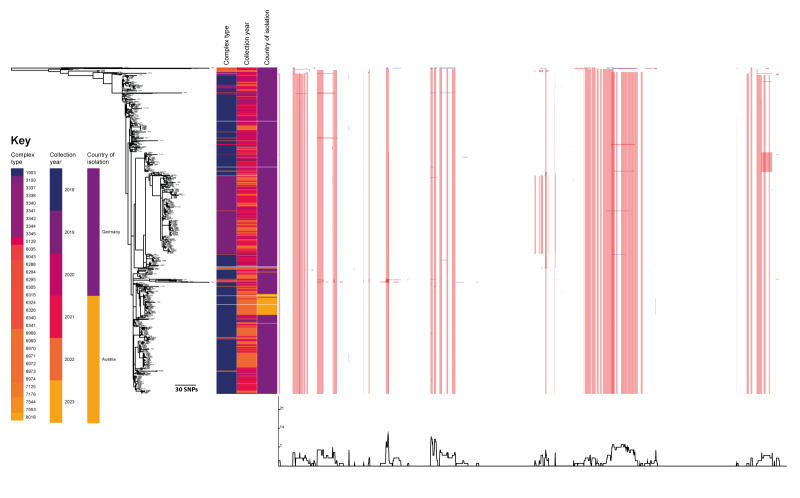
Recombinations within ST1299/*vanA* as identified by Gubbins

Recombinations were identified within the collection of ST1299/*vanA* (Figure 6), with SNP diversity still apparent, as seen by long branches. This suggests underlying genomic backbone diversity, rather than high genomic identity across ST1299/*vanA* masked only by recombination.

Recombination adjust phylogeny is shown on the left with tracks illustrating identified recombinations (in more than one genome in red and in single genomes in blue to the right). Identified by Gubbins version 3.3.0 [33]. Figure generated in Phandango [52].

Temporal modelling of the phylogeny suggests a most recent common ancestor of the clade in 1959 (95% CI: 1946–1972). The lack of data from that period prevents a precise analysis of ST1299/*vanA* 's origins.

## Discussion

We describe the path of the new VREfm strain ST1299/*vanA* during its spread from local via regional (2018) to cross-border level (2021) by using a combination of WGS and a large, cross-border strain collection. This collection initially included all cases known to the UHoR, then cases known to the German NRC, AGES, and MBR. By 2022, only eight further ST1299/*vanA* isolates had been reported regionally, specifically from two German cities (Erlangen and Hamburg), whereas 19 further isolates were seen internationally [18,21]. The earliest international case was detected in Denmark in 2017, differing by 77 alleles from isolates later found in Austria [20].

Our data show a rapid expansion of ST1299/*vanA* that started concomitantly in 2018 in Regensburg and another Bavarian city [17]. The cgMLST analysis revealed a polyclonal spread with 32 CTs detected throughout the study period. Of these, two subtypes – CT1903 (430 isolates) and later CT3109 (151 isolates) – made up 91.5% of isolates, whereas most other CT isolates were represented by single figures.

While only a few cases were seen during the first years, namely 2018 and 2019, rapid outbreak-related local, regional, and then national spread was observed, starting in 2020. Although only a small outbreak including four patients was detected in 2020, several VREfm outbreaks were reported during the COVID-19 pandemic [14,36-38]. The United States Centres for Disease Control and Prevention (US CDC) registered 14% more nosocomial VREfm cases than in 2019 [39]. Larger VREfm outbreaks were seen at UHoR in 2021 and 2022 (data not shown). Due to missing hospitalisation details from other participating centres, the impact of the pandemic is not quantifiable. Moreover, increased antibiotic consumption - most likely in severely ill COVID-19 patients - starting in 2020 may have promoted the spread of VREfm by promoting antibiotic selective pressure.

The first cases from our collection seen outside Bavaria were from the state of North Rhine-Westphalia, involving subtype CT1903, and in 2022 the state of North Rhine-Westphalia for CT3109. Moreover, the initial spread of these two CT differed geographically (CT1903 in southern Bavaria and North Rhine-Westphalia, CT3109 in Regensburg and northern Bavaria). Subtype CT3109 was also found in wastewater from Erlangen, Germany, strengthening a potential origin outside Regensburg [19]. No CT3109 isolates were detected in Austria between 2016 and 2022. As genome-oriented surveillance for VREfm is not required in Austria or Germany, we cannot exclude that other regions were also affected by VREfm ST1299/*vanA* [40]. However, recent literature from Bavaria does not report the detection of VREfm ST1299/*vanA* [41,42]. Moreover, the NRC recorded that simultaneous VREfm/*vanA* cases from northern Germany were predominantly ST80/CT1470 and ST117/CT929 [15].

Of all the ST1299/*vanA* cases at UHoR, 4.8% (22/460 isolates) were from BC. This may indicate that while ST1299/*vanA* has high transmission potential, it is not highly prone to develop to BSI compared with other STs [43,44]. With this hypothesis, given that 69 of 849 isolates (including one from Regensburg) from BSI sent to the NRC were identified as ST1299/*vanA* between 2018 and 2022, we assume that there may be a high number of unreported cases in southern Germany [15,45,46]. This is even more concerning as according to Ubeda et al. and Willems et al., VREfm colonisation precedes BSI and ST1299/*vanA* may thus become a clinical problem during further spread [47,48]. However, as WGS is becoming increasingly available in different institutions, some uncertainty remains regarding possible gaps in the NRC data [15]. Data from a large prevalence study performed in October 2020, however, shows that ST1299/*vanA* was not detected further north than Frankfurt am Main [49].

The first strains detected beyond the German border were found in Upper Austria during the analysis of clinical outbreaks [20]. Due to the immediate proximity of Upper Austria to the main region of ST1299/*vanA* endemicity in Germany and the late detection of the first ST1299/*vanA* cases, we hypothesise that patient transfer between countries may have caused the dissemination across the German-Austrian border. Similar events were described at the German-Dutch border, portraying the implications of globalisation on the spread of VREfm [50].

One important result of this study is the rate of nt substitution. Although it is known that VREfm evolves at a slow pace – 5 single nt polymorphisms per year according to Howden et al. – significant uncertainty remains about cgMLST's limitations for outbreak management and genomic surveillance [51]. This large early isolate collection shows high clonality, hindering differentiation between regional strains and true outbreak clusters (median allelic difference of nine in 2019). A shift in the median difference by six alleles per year was seen in 2021, when different strains caused large outbreaks in Regensburg. Thus, analysis of outbreaks involving emerging strains must be evaluated critically and in a regional epidemiological context. Moreover, the data show that cluster definitions of ≤ 20 alleles overestimate transmission events for endemic clonal lineages and could explain the conflicting results of previous studies on IPC [11,26]. In addition, WGS-based surveillance needs sufficient data on the epidemiological background to differentiate between transmissions and ‘young’ emerging strains [14].

Our attempt to identify the ancestors of ST1299/*vanA* detected that on a MLST level, seven PubMLST sequence types collected before 2018, including the worldwide predominant ST80, differed by 1–6 SNP (see Supplementary Table S2). Moreover, a Dutch ST80 isolate from 2015 from the international strain collection available in the PubMLST database was identified as closest related to the hybrid assembly of D6593. However, the common ancestor of our isolate collection is likely a clade from 1959. Among endemic strains detected before 2018, ST992, ST17 and ST18 were most similar to ST1299/*vanA*. Limitations of the analysis include arbitrary isolate selection from the UHoR database, a low number of strains used for comparison and missing data from 2011 to 2015.

Further limitations to our study include variability in screening criteria, possible underestimation of diversity due to sequencing of subcultures of only one colony, the voluntary and commonly outbreak-related submission of isolates to the NRC, missing WGS-based surveillance data from other hospitals and the cross-border strain collection in Upper Austria.

## Conclusion

Our unique strain collection demonstrates that the recent emergence of ST1299/*vanA* and outbreak-mediated propagation both led to high local and overall clonality. Tracing the transmission chains of an emerging strain remains challenging. Moreover, the ability of different CTs to move from local and regional dominance to a cross-border level varies. Thus, genome-based surveillance is needed at a national and international level to promptly identify emerging epidemic strains, and to enable the prevention of comparable spreads, the correct identification of VREfm outbreaks and possibly even to achieve the missing international consensus regarding IPC.

## Data Availability

Data have been deposited in the NCBI database under BioProject accession numbers: PRJNA1263826; PRJNA1263801; PRJNA1263828.

## Supplementary Data

205000 Supplementary Material

## References

[1] Hammerum AM. Enterococci of animal origin and their significance for public health. Clin Microbiol Infect. 2012;18(7):619-25. 10.1111/j.1469-0691.2012.03829.x 22487203

[2] Verway M Brown KA Marchand-Austin A Diong C Lee S Langford B Prevalence and Mortality Associated with Bloodstream Organisms: a Population-Wide Retrospective Cohort Study. J Clin Microbiol. 2022;60(4):e0242921. 10.1128/jcm.02429-21 35254101 PMC9020345

[3] Eichel VM Last K Brühwasser C von Baum H Dettenkofer M Götting T Epidemiology and outcomes of vancomycin-resistant enterococcus infections: a systematic review and meta-analysis. J Hosp Infect. 2023;141:119-28. 10.1016/j.jhin.2023.09.008 37734679

[4] Cassini A Högberg LD Plachouras D Quattrocchi A Hoxha A Simonsen GS Attributable deaths and disability-adjusted life-years caused by infections with antibiotic-resistant bacteria in the EU and the European Economic Area in 2015: a population-level modelling analysis. Lancet Infect Dis. 2019;19(1):56-66. 10.1016/S1473-3099(18)30605-4 30409683 PMC6300481

[5] Correa-Martínez CL Schuler F Kampmeier S. Sex differences in vancomycin-resistant enterococci bloodstream infections-a systematic review and meta-analysis. Biol Sex Differ. 2021;12(1):36. 10.1186/s13293-021-00380-5 34001270 PMC8130152

[6] Werner G Neumann B Weber RE Kresken M Wendt C Bender JK Thirty years of VRE in Germany - "expect the unexpected": The view from the National Reference Centre for Staphylococci and Enterococci. Drug Resist Updat. 2020;53:100732. 10.1016/j.drup.2020.100732 33189998

[7] World Health Organization (WHO). WHO publishes list of bacteria for which new antibiotics are urgently needed. Geneva: WHO; 2017. Available from: https://www.who.int/news/item/27-02-2017-who-publishes-list-of-bacteria-for-which-new-antibiotics-are-urgently-needed

[8] European Centre for Disease Prevention and Control (ECDC) and World Health Organization European Region. Antimicrobial resistance surveillance in Europe 2023–2021 data. Stockholm: ECDC; 2023. Available from: https://www.ecdc.europa.eu/en/publications-data/antimicrobial-resistance-surveillance-europe-2023-2021-data

[9] Correa-Martínez CL Jurke A Schmitz J Schaumburg F Kampmeier S Mellmann A. Molecular Epidemiology of Vancomycin-Resistant Enterococci Bloodstream Infections in Germany: A Population-Based Prospective Longitudinal Study. Microorganisms. 2022;10(1):130. 10.3390/microorganisms10010130 35056579 PMC8777844

[10] Bavarian Food and Health Safety Authority (LGL). BARDa – Resistenzlage in Bayern. [BARDa – Antimicrobial resistance situation in Bavaria]. Munich: LGL. [Accessed: 22 May 2024]. German. Available from: https://www.lgl.bayern.de/gesundheit/infektionsschutz/barda/barda_interaktiv.htm

[11] Vehreschild MJGT Haverkamp M Biehl LM Lemmen S Fätkenheuer G. Vancomycin-resistant enterococci (VRE): a reason to isolate? Infection. 2019;47(1):7-11. 10.1007/s15010-018-1202-9 30178076

[12] Johnstone J Shing E Saedi A Adomako K Li Y Brown KA Discontinuing Contact Precautions for Vancomycin-Resistant Enterococcus (VRE) Is Associated With Rising VRE Bloodstream Infection Rates in Ontario Hospitals, 2009-2018: A Quasi-experimental Study. Clin Infect Dis. 2020;71(7):1756-9. 10.1093/cid/ciaa009 31922536

[13] Caplunik-Pratsch A Kieninger B Donauer VA Brauer JM Meier VMK Seisenberger C Introduction and spread of vancomycin-resistant Enterococcus faecium (VREfm) at a German tertiary care medical center from 2004 until 2010: a retrospective whole-genome sequencing (WGS) study of the molecular epidemiology of VREfm. Antimicrob Resist Infect Control. 2024;13(1):20. 10.1186/s13756-024-01379-4 38355509 PMC10865517

[14] Rath A Kieninger B Caplunik-Pratsch A Concerning emergence of a new vancomycin-resistant Enterococcus faecium strain ST1299/CT1903/vanA at a tertiary university centre in South-Germany. J Hosp Infect. 2023. 37852539 10.1016/j.jhin.2023.10.008

[15] Fischer MA, Bender JK, Kriebel N, et al. Eigenschaften, Häufigkeit und Verbreitung von Vancomycin-resistenten Enterokokken in Deutschland – Update. [Characteristics, Frequency, and Spread of Vancomycin-Resistant Enterococci in Germany – Update]. Epid Bull. 2023;28:3-17. German. Available from: https://edoc.rki.de/handle/176904/11224

[16] Jochim-Vukosavic A Schwab F Knegendorf L Schlüter D Bange FC Ebadi E Epidemiology and infection control of vancomycin-resistant enterococci at a German university hospital: A three-year retrospective cohort study. PLoS One. 2024;19(2):e0297866. 10.1371/journal.pone.0297866 38408053 PMC10896503

[17] Trautmannsberger I Kolberg L Meyer-Buehn M Huebner J Werner G Weber R Epidemiological and genetic characteristics of vancomycin-resistant Enterococcus faecium isolates in a University Children’s Hospital in Germany: 2019 to 2020. Antimicrob Resist Infect Control. 2022;11(1):48. 10.1186/s13756-022-01081-3 35279207 PMC8917738

[18] Valenza G Eisenberger D Voigtländer S Alsalameh R Gerlach R Koch S Emergence of novel ST1299 vanA lineages as possible cause for the striking rise of vancomycin resistance among invasive strains of Enterococcus faecium at a German university hospital. Microbiol Spectr. 2023;11(6):e0296223. 10.1128/spectrum.02962-23 37905844 PMC10848474

[19] Valenza G Eisenberger D Esse J Held J Lehner-Reindl V Plaumann P-L High prevalence of the recently identified clonal lineage ST1299/CT3109 vanA among vancomycin-resistant Enterococcus faecium strains isolated from municipal wastewater. MSphere. 2024;9(9):e0039624. 10.1128/msphere.00396-24 39189779 PMC11423563

[20] Cabal A Hörtenhuber A Halabi M Kerschner H Salaheddin Y Ruppitsch W. First detection of the emerging vancomycin-resistant Enterococcus faecium vanA-ST1299-CT1903 in Austria. Clin Microbiol Infect. 2024;30(12):1609-12. 10.1016/j.cmi.2024.08.010 39163916

[21] Paul K Merabishvili M Hazan R Christner M Herden U Gelman D Bacteriophage Rescue Therapy of a Vancomycin-Resistant Enterococcus faecium Infection in a One-Year-Old Child following a Third Liver Transplantation. Viruses. 2021;13(9):1785. 10.3390/v13091785 34578366 PMC8472888

[22] The Public Health Agency of Sweden and National Veterinary Institute. Swedres-Svarm, 2022. Sales of antibiotics and occurrence of resistance in Sweden. Stockholm: The Public Health Agency of Sweden; 2022. Available from: https://www.sva.se/media/s3ggt1ny/swedres-svarm-2022-edit-230808.pdf

[23] von Elm E Altman DG Egger M Pocock SJ Gøtzsche PC Vandenbroucke JP STROBE Initiative. Strengthening the Reporting of Observational Studies in Epidemiology (STROBE) statement: guidelines for reporting observational studies. BMJ. 2007;335(7624):806-8. 10.1136/bmj.39335.541782.AD 17947786 PMC2034723

[24] Naserpour Farivar T Najafipour R Johari P Aslanimehr M Peymani A Jahani Hashemi H Development and evaluation of a Quadruplex Taq Man real-time PCR assay for simultaneous detection of clinical isolates of Enterococcus faecalis, Enterococcus faecium and their vanA and vanB genotypes. Iran J Microbiol. 2014;6(5):335-40. 25848524 PMC4385574

[25] Souvorov A Agarwala R Lipman DJ. SKESA: strategic k-mer extension for scrupulous assemblies. Genome Biol. 2018;19(1):153. 10.1186/s13059-018-1540-z 30286803 PMC6172800

[26] de Been M Pinholt M Top J Bletz S Mellmann A van Schaik W Core Genome Multilocus Sequence Typing Scheme for High- Resolution Typing of Enterococcus faecium. J Clin Microbiol. 2015;53(12):3788-97. 10.1128/JCM.01946-15 26400782 PMC4652124

[27] Homan WL Tribe D Poznanski S Li M Hogg G Spalburg E Multilocus sequence typing scheme for Enterococcus faecium. J Clin Microbiol. 2002;40(6):1963-71. 10.1128/JCM.40.6.1963-1971.2002 12037049 PMC130786

[28] Letunic I Bork P. Interactive Tree of Life (iTOL) v6: recent updates to the phylogenetic tree display and annotation tool. Nucleic Acids Res. 2024;52(W1):W78-82. 10.1093/nar/gkae268 38613393 PMC11223838

[29] Hasman H Clausen PTLC Kaya H Hansen F Knudsen JD Wang M LRE-Finder, a Web tool for detection of the 23S rRNA mutations and the optrA, cfr, cfr(B) and poxtA genes encoding linezolid resistance in enterococci from whole-genome sequences. J Antimicrob Chemother. 2019;74(6):1473-6. 10.1093/jac/dkz092 30863844

[30] Jolley KA Bray JE Maiden MCJ. Open-access bacterial population genomics: BIGSdb software, the PubMLST.org website and their applications. Wellcome Open Res. 2018;3:124. 10.12688/wellcomeopenres.14826.1 30345391 PMC6192448

[31] Zhou Z Alikhan NF Sergeant MJ Luhmann N Vaz C Francisco AP GrapeTree: visualization of core genomic relationships among 100,000 bacterial pathogens. Genome Res. 2018;28(9):1395-404. 10.1101/gr.232397.117 30049790 PMC6120633

[32] Didelot X Croucher NJ Bentley SD Harris SR Wilson DJ. Bayesian inference of ancestral dates on bacterial phylogenetic trees. Nucleic Acids Res. 2018;46(22):e134. 10.1093/nar/gky783 30184106 PMC6294524

[33] Croucher NJ Page AJ Connor TR Delaney AJ Keane JA Bentley SD Rapid phylogenetic analysis of large samples of recombinant bacterial whole genome sequences using Gubbins. Nucleic Acids Res. 2015;43(3):e15. 10.1093/nar/gku1196 25414349 PMC4330336

[34] Seemann T. Snippy: fast bacterial variant calling from NGS reads. [Accessed: 23 Sep 2024]. Available from: https://github.com/tseemann/snippy.

[35] Meier-Kolthoff JP Göker M. TYGS is an automated high-throughput platform for state-of-the-art genome-based taxonomy. Nat Commun. 2019;10(1):2182. 10.1038/s41467-019-10210-3 31097708 PMC6522516

[36] Rath A Kieninger B Mirzaliyeva N Schmid S Mester P Schneider-Brachert W. The genome-oriented surveillance of vancomycin-resistant enterococci shows a clear misclassification of nosocomial transmission events. Clin Microbiol Infect. 2024;30(8):1086-8. 10.1016/j.cmi.2024.04.010 38663654

[37] Kampmeier S Tönnies H Correa-Martinez CL Mellmann A Schwierzeck V. A nosocomial cluster of vancomycin resistant enterococci among COVID-19 patients in an intensive care unit. Antimicrob Resist Infect Control. 2020;9(1):154. 10.1186/s13756-020-00820-8 32962759 PMC7506805

[38] Rathod SN Bardowski L Tse I Churyla A Fiehler M Malczynski M Vancomycin-resistant Enterococcus outbreak in a pre- and post-cardiothoracic transplant population: Impact of discontinuing multidrug-resistant organism surveillance during the coronavirus disease 2019 pandemic. Transpl Infect Dis. 2022;24(6):e13972. 10.1111/tid.13972 36169219

[39] Centers for Disease Control and Prevention (CDC). COVID-19: U.S. Impact on Antimicrobial Resistance, Special Report 2022. Atlanta: CDC; 2022. Available from: https://www.cdc.gov/antimicrobial-resistance/media/pdfs/covid19-impact-report-508.pdf

[40] Kommission für Krankenhaushygiene und Infektionsprävention (KRINKO) beim Robert Koch-Institut. Hygienemaßnahmen zur Prävention der Infektion durch Enterokokken mit speziellen Antibiotikaresistenzen. [Hygiene Measures for Preventing Infections Caused by Enterococci with Specific Antibiotic Resistances]. Bundesgesundheitsblatt Gesundheitsforschung Gesundheitsschutz. 2018;61(10):1310-61. 10.1007/s00103-018-2811-2 30229318

[41] Eisenberger D Tuschak C Werner M Bogdan C Bollinger T Hossain H Whole-genome analysis of vancomycin-resistant Enterococcus faecium causing nosocomial outbreaks suggests the occurrence of few endemic clonal lineages in Bavaria, Germany. J Antimicrob Chemother. 2020;75(6):1398-404. 10.1093/jac/dkaa041 32083651

[42] Neumann B Bender JK Maier BF Wittig A Fuchs S Brockmann D Comprehensive integrated NGS-based surveillance and contact-network modeling unravels transmission dynamics of vancomycin-resistant enterococci in a high-risk population within a tertiary care hospital. PLoS One. 2020;15(6):e0235160. 10.1371/journal.pone.0235160 32579600 PMC7314025

[43] Piezzi V Wassilew N Atkinson A D’Incau S Kaspar T Seth-Smith HM Nosocomial outbreak of vancomycin-resistant Enterococcus faecium (VRE) ST796, Switzerland, 2017 to 2020. Euro Surveill. 2022;27(48):2200285. 10.2807/1560-7917.ES.2022.27.48.2200285 36695463 PMC9716646

[44] Wassilew N Seth-Smith HM Rolli E Fietze Y Casanova C Führer U Outbreak of vancomycin-resistant Enterococcus faecium clone ST796, Switzerland, December 2017 to April 2018. Euro Surveill. 2018;23(29):1800351. 10.2807/1560-7917.ES.2018.23.29.1800351 30043725 PMC6152203

[45] Klare I, Bender JK, Marktwart R, Reuss A, Abu Sin M, Eckmanns T, et al. Eigenschaften, Häufigkeit und Verbreitung von Vancomycin-resistenten Enterokokken in Deutschland – Update 2017/2018. [Characteristics, Frequency, and Spread of Vancomycin-Resistant Enterococci in Germany – Update 2017/2018]. Epid Bull. 2019;37:365-372. German. 10.25646/6236.2

[46] Weber RE, Bender JK, Werner G, Noll I, Abu Sin M, Eckmanns T. Eigenschaften, Häufigkeit und Verbreitung von Vancomycin-resistenten Enterokokken in Deutschland – Update 2019/2020. [Characteristics, Frequency, and Spread of Vancomycin-Resistant Enterococci in Germany – Update 2019/2020]. Epid Bull. 2021;27:32-42. German."https://www.rki.de/DE/Aktuelles/Publikationen/Epidemiologisches-Bulletin/2021/27_21.pdf?__blob=publicationFile&v=1" 10.25646/8710

[47] Ubeda C Taur Y Jenq RR Equinda MJ Son T Samstein M Vancomycin-resistant Enterococcus domination of intestinal microbiota is enabled by antibiotic treatment in mice and precedes bloodstream invasion in humans. J Clin Invest. 2010;120(12):4332-41. 10.1172/JCI43918 21099116 PMC2993598

[48] Willems RPJ van Dijk K Vehreschild MJGT Biehl LM Ket JCF Remmelzwaal S Incidence of infection with multidrug-resistant Gram-negative bacteria and vancomycin-resistant enterococci in carriers: a systematic review and meta-regression analysis. Lancet Infect Dis. 2023;23(6):719-31. 10.1016/S1473-3099(22)00811-8 36731484

[49] Schneider W, Rath A, Eichner A, et al. P1294 - Population snapshot of vancomycin-resistant enterococci in central Europe. Presented at Congress of the European Society of Clinical Microbiology and Infectious Diseases (ESCMID) Global. Barcelona, Spain, 2024.

[50] Cimen C Berends MS Bathoorn E Lokate M Voss A Friedrich AW Vancomycin-resistant enterococci (VRE) in hospital settings across European borders: a scoping review comparing the epidemiology in the Netherlands and Germany. Antimicrob Resist Infect Control. 2023;12(1):78. 10.1186/s13756-023-01278-0 37568229 PMC10422769

[51] Howden BP Holt KE Lam MM Seemann T Ballard S Coombs GW Genomic insights to control the emergence of vancomycin-resistant enterococci. MBio. 2013;4(4):e00412-3. 10.1128/mBio.00412-13 23943759 PMC3747580

[52] Hadfield J Croucher NJ Goater RJ Abudahab K Aanensen DM Harris SR. Phandango: an interactive viewer for bacterial population genomics. Bioinformatics. 2018;34(2):292-3. 10.1093/bioinformatics/btx610 29028899 PMC5860215

