# Supplementary material for: Spread of the novel vancomycin-resistant Enterococcus faecium strain ST1299/vanA from local level in Germany to cross-border level in Austria, 2018 to 2022
Source: Euro Surveill. 2025 May 22;30(20):2400389. doi: 10.2807/1560-7917.ES.2025.30.20.2400389 (PMC12105089; doi:10.2807/1560-7917.ES.2025.30.20.2400389)
Supplement: Supplementary Material [file 24-00389_RATH_Supplement.pdf]

This supplementary material is hosted by *Eurosurveillance* as supporting information alongside the article ‚Spread of the novel vancomycin-resistant *Enterococcus faecium* strain ST1299/vanA from local level in Germany to cross-border level in Austria, 2018 to 2022', on behalf of the authors, who remain responsible for the accuracy and appropriateness of the content. The same standards for ethics, copyright, attributions and permissions as for the article apply. Supplements are not edited by *Eurosurveillance* and the journal is not responsible for the maintenance of any links or email addresses provided therein.

Supplementary Table S1. Overview of isolates used for the phylogeny analysis and the phylogram

ID	ST	CT	SNP*	alleles to Ref by cgMLST	Year of collection
U4758	1299	1903	110	15	2018
U2509	17	3256	4831	355	2008
U3688	17	900	5376	372	2016
U1932	17	3251	4905	356	2007
U18758	18	280	4424	304	2019
U20873	18	3047	4250	404	2011
U2381	18	1898	5088	356	2020
U685	18	3276	5970	400	2005
U3384	78	894	5841	376	2017
U18523	78	5584	5948	379	2017
U3464	80	1013	5689	376	2016
U5266	80	1552	7892	303	2017
U3525	117	929	5865	367	2017
U3726	117	469	6566	387	2016
U2925	117	24	6932	362	2010
U8314	186	3263	5279	393	2010
U2338	192	10	5177	388	2008
U3604	192	152	5220	401	2016
U2542	202	2665	5285	402	2008
U1758	208	3262	5215	399	2005
U18689	721	1573	6041	355	2018
U708	780	3245	5256	402	2006
U18268	992	7179	6495	273	2016
U3372	1478	929	6683	375	2017
U12702	1478	2967	6743	375	2017

***SNP to Ref**

< 4000 | 4000-4999 | 5000-5999 | 6000-6999 | > 7000

Supplementary Table S2. Overview of isolates in PubMLST nearest matching ST1299 that were collected in or prior to 2018

ST	atpA	ddl	gdh	purK	gyd	pstS	adk	No. of isoaltes in PubMLST	Country	Year	Mutation	
ST1299	9	1	1	97	12	1	1	Reference			Gen locus	No. of SNP
ST80	9	1	1	1	12	1	1	372	multiple	--	purK	1
ST1032	9	1	1	44	12	1	1	1	United Kingdom	2011	purK	2
ST1331	9	1	1	98	12	1	1	1	The Netherlands	2017	purK	1
ST1353	9	1	1	95	12	1	1	1	The Netherlands	2017	purK	2
ST1623	9	1	1	2	12	1	1	1	Australia	2018	purK	2
ST2451	9	1	1	169	12	1	1	1	New Zealand	2018	purK	2
ST1903	9	3	1	97	12	1	1	1	Denmark	2016	ddl	6

Source: https://pubmlst.org/bigsdb?db=pubmlst_efaecium_seqdef&page=profiles , accessed 18.10.2024, 15:00 o'clock;
*isolate from UHoR; *grey* – differing allele
